# The association between 2017 American College of Cardiology/American Heart Association guideline for hypertension and neonatal outcomes in Kenya: a retrospective study

**DOI:** 10.1186/s41182-025-00724-6

**Published:** 2025-03-27

**Authors:** Mami Hitachi, Kazuchiyo Miyamichi, Sumihisa Honda, Violet Wanjihia, Samson Muuo Nzou, Satoshi Kaneko

**Affiliations:** 1 Department of Ecoepidemiology, Institute of Tropical Medicine, Nagasaki University, Nagasaki, Japan; 2 Kenya Research Station, Institute of Tropical Medicine, Nagasaki University, Nagasaki, Japan; 3 Department of Nursing, Nagasaki University Graduate School of Biomedical Sciences, Nagasaki, Japan; 4 Centre for Public Health Research, Kenya Medical Research Institute, Nairobi, Kenya; 5 Centre for Microbiology Research, Kenya Medical Research Institute, Nairobi, Kenya

**Keywords:** Adverse neonatal outcome, Hypertension, Pregnancy, Preterm birth, Low birth weight, Low-resource setting

## Abstract

**Background:**

Hypertension in pregnancy serves to screen for adverse perinatal outcomes. In 2017, the American College of Cardiology and American Heart Association recommended a new blood pressure category with lower hypertension thresholds, excluding pregnancy. This study aimed to explore the association between the 2017 redefined blood pressure categories in pregnancy and neonatal outcomes such as preterm birth and low birth weight.

**Methods:**

This retrospective study used electronic records of the Maternal and Child Health Handbook registered by the Women and Infant Registration System. All women who had at least one antenatal care visit and delivery between January 2017 and April 2020 and between May and December 2022 were included in the study. A birth of less than 37 weeks was defined as preterm delivery. LBW was identified based on a newborn’s birthweight of less than 2500 g. The maximum blood pressure across all antenatal care visits was classified based on the newly recommended criteria. A generalized linear model with binomial distribution and logit link function was used to evaluate the association between new blood pressure categories and neonatal outcomes at different levels of health facilities.

**Results:**

We analyzed data from 825 women. Of these, the prevalence was 13.7% for elevated blood pressure, 15.2% for stage 1 hypertension, 4.5% for non-severe stage 2 hypertension and 1.2% for severe stage 2 hypertension. For lower-level facilities, no significant associations were identified between the redefined blood pressure category and preterm birth or low birthweight. At higher-level facilities, preterm birth was only significantly associated with severe stage 2 hypertension (adjusted odds ratio:10.94; 95% confidence interval:1.08–110.93; *P* = 0.04) and low birthweight showed no association with the redefined category.

**Conclusion:**

This study revealed no association between redefined lower blood pressure threshold and preterm birth and low birthweight in under-resourced settings. However, previous studies in well-resourced countries with larger sample sizes also reported a significant association. Therefore, further investigations are required.

## Introduction

Hypertension during pregnancy is a major public health concern due to its association with adverse maternal and perinatal outcomes, particularly in low- and middle-income countries (LMICs) [1–3]. Hypertensive disorders in pregnancy include several distinct clinical entities. They are classified into four types: chronic hypertension, gestational hypertension, preeclampsia, and superimposed preeclampsia. Chronic hypertension is defined as hypertension present before pregnancy or diagnosed before 20 weeks of gestation. Gestational hypertension is characterized by new-onset hypertension after 20 weeks of gestation. Preeclampsia is hypertension after 20 weeks with proteinuria, organ damage, or uteroplacental dysfunction. Superimposed preeclampsia is defined as hypertension accompanied by proteinuria, worsening of blood pressure control, and/or HELLP syndrome (Hemolysis, Elevated Liver enzymes, Low Platelet count) [4, 5].

Traditionally, hypertension in pregnancy has long been defined as at least one systolic blood pressure (sBP) ≥ 140 mm Hg or at least one diastolic blood pressure (dBP) ≥ 90 mm Hg, or both [6]. These criteria serve as a screening test to identify pregnant women and newborns with a high risk of maternal, fetal, and neonatal complications.

In 2017, new blood pressure (BP) categories with lower hypertension thresholds were recommended by the American College of Cardiology (ACC) and American Heart Association (AHA), although notably excluding pregnancy. These revised categories include normal blood pressure (sBP < 120 mm Hg and dBP < 80 mm Hg), elevated blood pressure (sBP 120–129 mm Hg and dBP < 80 mm Hg), stage 1 hypertension (sBP 130–139 mm Hg or dBP 80–89 mm Hg, or both), non-severe stage 2 hypertension (sBP 140–159 mm Hg or dBP 90–109 mm Hg, or both) and severe stage 2 hypertension (sBP ≥ 160 mm Hg or dBP ≥ 110 mm Hg, or both) [7]. The adoption of the new BP categories could reclassify numerous women who are currently not deemed hypertensive, potentially doubling the prevalence of hypertension in women of reproductive age [8].

Previous studies revealed that newly defined hypertension, stage 1 hypertension by the 2017 ACC/AHA guideline, is correlated with preeclampsia as an adverse maternal outcome [9–16]. However, its relationship with neonatal outcomes remains inconsistent. Some studies have revealed an increased risk of redefined categories, stage 1 hypertension, for poor neonatal outcomes including preterm birth, LBW, and small for gestational age [3, 9, 10, 14, 17–20]. In contrast, other studies have not demonstrated the association between stage 1 hypertension and neonatal outcomes [11, 21, 22]. Therefore, further research is necessary to validate these findings across diverse populations and evaluate these lower thresholds’ clinical applicability as diagnostic criteria [8, 13, 16].

Most previous studies have been conducted in well-resourced settings, leaving a gap in evidence from LMICs. Preterm birth and low birth weight remain critical public health issues in these regions. They contribute significantly to neonatal deaths, which account for about half of all under-five deaths in LMICs [23]. Furthermore, the impact of preterm birth and LBW extends beyond infancy, leading to long-term effects that can burden individuals and hinder societal progress [24]. Given these significant health burdens, there is a pressing need to evaluate the potential of 2017 ACC/AHA guidelines to identify pregnancies at risk for adverse outcomes. Therefore, this study aims to examine the association between the incidence of preterm birth and LBW among pregnant women newly classified as hypertensive under the 2017 ACC/AHA guidelines in resource-limited settings.

## Methods

### Study design and participants

We conducted a retrospective study using information from the electronic maternal and child health (MCH) booklet recorded by Women and Infant Registration (WIRE), a cloud-based custom maternal and child health registration system developed by our research team and installed at rural health facilities in Kwale County, Kenya. Trained facility staff registered data using WIRE computers located at each health facility. Upon initial registration, individual WIRE ID numbers were generated and connected to records on antenatal care (ANC) visits, deliveries, and postnatal care (PNC) visits for growth monitoring and immunizations. Details of the WIRE system have been reported previously [25]. All women with at least one ANC visit and delivery at a WIRE-participating facility during the study period were recruited. Women who were HIV positive or had multiple births were excluded because they could be potential confounders for the association between maternal hypertension and adverse perinatal outcomes [26–28].

### Data collection

The first WIRE phase registered data between January 2017 and April 2020 excluding 150 days of nurses’ strike (from June to November 2017) [26]. After 2 years of system adjustments and preparation for site expansion, the second phase was conducted from May 2022 to December 2022. Registered data were extracted from the WIRE database for this study. The first phase was delivered at six MCH facilities: Kwale Sub-county Hospital, Kinango Sub-county Hospital, Diani Health Clinic, Mwaluphamba Dispensary, Mwachinga Dispensary, and Vyongwani Dispensary. The second phase was introduced the WIRE at nine MCH facilities: five facilities, excluding Diani Health Clinic from the first phase, and four additional health facilities as Chitsanze Dispensary, Dumbule Dispensary, Kizibe Dispensary and Miatsani Dispensary. Records of ANC (BP, hemoglobin level, preventive services, gestational age at the first visit, number and date of visit and maternal age and weight) and delivery (newborns’ sex and birthweight and date of delivery) were exported from the WIRE database. The maximum value of registered sBP and dBP across all ANC visits for each woman were classified based on the ACC/AHA new criteria: normal BP (sBP < 120 mm Hg and dBP < 80 mm Hg), elevated BP (sBP 120–129 mm Hg and dBP < 80 mm Hg), stage 1 hypertension (sBP 130–139 mm Hg or dBP 80–89 mm Hg, or both), non-severe stage 2 hypertension (sBP 140–159 mm Hg or dBP 90–109 mm Hg, or both), and severe stage 2 hypertension (sBP ≥ 160 mm Hg or dBP ≥ 110 mm Hg, or both). Hence, no distinction was made between chronic and gestational hypertension. Anemia was defined as a minimum hemoglobin level of < 11 g/dl. For preventive services, receiving of the following items were identified as variables (0/1): iron and folic acid supplementation, malaria prophylaxis and long-lasting insecticide treated net (LLITN), Tetanus Diphtheria (TD) vaccination and deworming. The gestational age at the first ANC visit was calculated by the health staff based on the women’s recall of the last menstrual period. The total number of ANC visits was categorized as less or more than four times. This is because more than four ANC visits are medically recommended [29]. Maternal age was classified by women aged less than or equal to 19 years, from 20 to 34 years and over 35 years because adolescent pregnancy and advanced maternal age are known as risk factors for gestational hypertension [30, 31]. Obesity was identified if booking weight, the first recorded weight during antenatal care, was above 90 kg. That body mass index could not be calculated to define obesity because women’s height had not been measured. A previous study presented booking weight as a confounder to evaluate the association between maternal BP and adverse maternal outcomes [32]. The level of MCH facilities was classified higher for hospitals positioned as referral facilities and lower for dispensaries and health centers.

### Outcome

The outcome assessed in this study was the incidence of preterm birth and LBW. Preterm birth was defined as a delivery with the gestational age of less than 37 weeks. Gestational age at delivery was calculated using that of the first ANC visit. LBW was identified based on a newborn’s birthweight of less than 2500 g.

### Statistical analysis

Descriptive statistics were used to summarize the proportion of each BP category based on participant characteristics. A generalized linear model (GLM) with binomial distribution and logit link function (logistic regression model) was used to assess the association between BP category and adverse perinatal outcomes, such as preterm birth and LBW. The odds ratio (OR) was calculated by elevated BP, stage 1 hypertension and non-severe and severe stage 2 hypertension applying normal BP as a reference. For the OR of LBW, non-severe and severe stage 2 hypertension were combined because no women with severe stage 2 hypertension had LBW. The best model was chosen by backward stepwise model selection using Akaike’s information criterion (AIC). The full model for preterm birth included variables of maternal BP, anemia, obesity and age, preventive services and newborn sex. For LBW, the gestational week at delivery was added to the full model for preterm birth. The analysis was separately conducted at the MCH facility level (higher or lower) because high-level facilities are referral hospitals to care for obstetric complications. The crude odds ratio (COR) and adjusted odds ratio (AOR) with 95% confidence interval (CI) were reported. P-value less than or equal to 0.05 was considered statistically significant. All statistical analyses were performed using STATA 14 (StataCorp LLC, college Station, TX, USA).

### Ethical consideration

This study was approved by the Kenya Medical Research Institute (KEMRI) Scientific Ethical Review Unit (SERU) (KEMRI/SERU/3746 for the first phase and KEMRI/SERU/7/3/1 for the second phase) and the Institutional Review Board of the Institute of Tropical Medicine, Nagasaki University (140117120 for the first phase and 200910246 for the second phase). We explained WIRE to all participants before enrolling. Data registration was performed only after informed consent was obtained from the participants.

## Result

### Participants characteristics

**Fig. 1:**
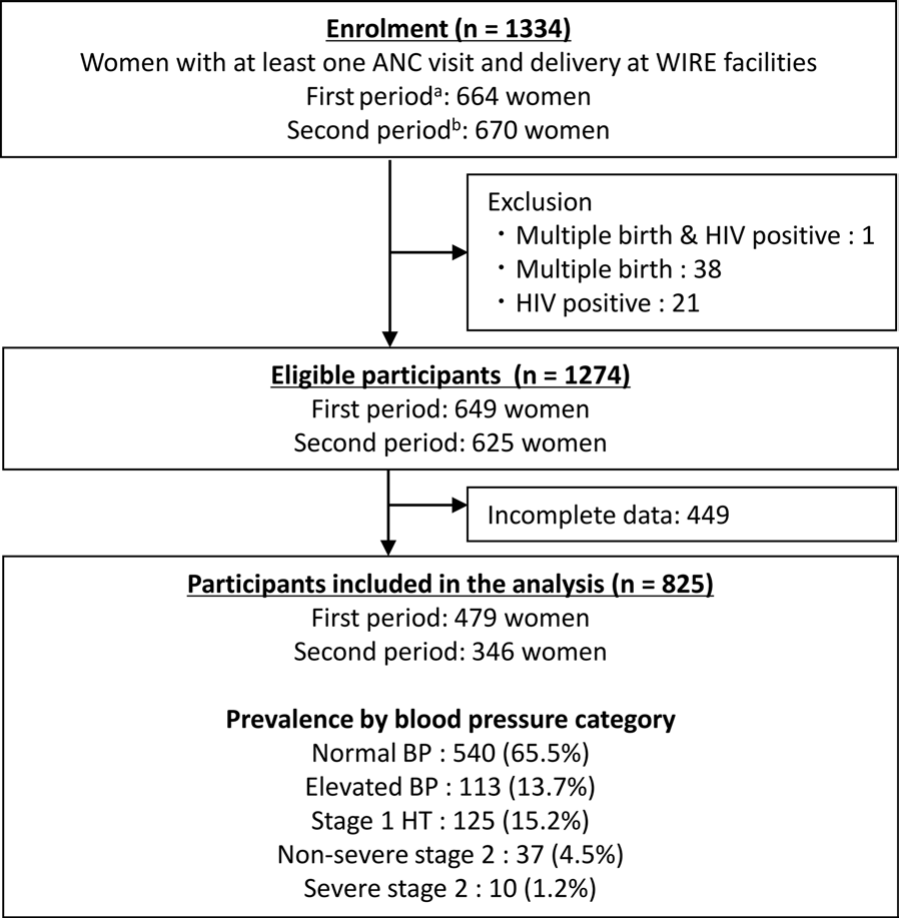
Flow diagram of the enrolled participants and study procedures. *BP* blood pressure, *HT* hypertension. **a** First period is between January 2017 and April 2020 at six facilities. **b** Second period is between May 2022 and December 2023 at nine facilities

A total of 1334 women received ANC services at least once and delivered at WIRE-participating facilities: 664 women between January 2017 and April 2020 (excluding the nurses’ strike period) and 670 women between May 2022 and December 2023. Finally, 825 women were eligible for the analysis after the exclusion of 60 women with multiple births or HIV positive and 449 women with incomplete data (Fig. 1). Overall, about one-third of the participants (34.5%) were classified into any category of abnormality based on the newly recommended criteria: 113 women had elevated BP (13.7%), 125 women had stage 1 hypertension (15.2%), 37 women had non-severe stage 2 hypertension (4.5%) and10 women had severe stage 2 hypertension (1.2%) (Table 1). Approximately one out of four women was high-risk pregnancy as an adolescent or advanced age (25.5%). The prevalence of obesity was low (4.2%), whereas that of anemia was high (61.3%). Only one out of five (21.6%) women underwent ANC more than four times. The coverage of medication for anemia and malaria was relatively high (96.4% and 84.2%, respectively), although it was low for deworming and Tetanus–Diphtheria injection (25.2% and 32.4%, respectively). In addition, only 13.7% of the women received an Insecticide Treated Net. The proportion of higher and lower MCH facility levels was almost equal (49.1% vs. 50.9%). Those of sex for newborns (female/male) are also similar with each other (47.3% vs. 52.7%).

**Table 1:** Characteristics of participants

Variables	Participants (*N* = 825)									
	Normal BP	(%)	Elevated BP	(%)	Stage1 HT	(%)	Non-severe stage 2 HT	(%)	Severe stage 2 HT	(%)
Maternal age (years)										
≤ 19	57	(10.6)	21	(18.6)	10	(8.0)	2	(5.4)	1	(10.0)
20–34	410	(75.9)	73	(64.6)	99	(79.2)	26	(70.3)	7	(70.0)
≥ 35	73	(13.5)	19	(16.8)	16	(12.8)	9	(24.3)	2	(20.0)
Obesity										
No	526	(97.4)	105	(92.9)	116	(92.8)	33	(89.2)	10	(100.0)
Yes	14	(2.6)	8	(7.1)	9	(7.2)	4	(10.8)	0	(0.0)
Anemia										
No	201	(37.2)	47	(41.6)	50	(40.0)	18	(48.7)	3	(30.0)
Yes	339	(62.8)	66	(58.4)	75	(60.0)	19	(51.3)	7	(70.0)
Number of ANC visit										
< 4	433	(80.2)	82	(72.6)	94	(75.2)	29	(78.4)	9	(90.0)
≥ 4	107	(19.8)	31	(27.4)	31	(24.8)	8	(21.6)	1	(10.0)
Preventive service										
Iron and folic acid supplementation										
No	27	(5.0)	2	(1.7)	0	(0.0)	0	(0.0)	1	(10.0)
Yes	513	(95.0)	111	(98.3)	125	(100.0)	37	(100.0)	9	(90.0)
Malaria prophylaxis										
No	86	(15.9)	13	(11.5)	24	(19.2)	6	(16.2)	1	(10.0)
Yes	454	(84.1)	100	(88.5)	101	(80.8)	31	(83.8)	9	(90.0)
Insecticide treated net										
No	464	(85.9)	104	(92.0)	104	(83.2)	33	(89.2)	7	(70.0)
Yes	76	(14.1)	9	(8.0)	21	(16.8)	4	(10.8)	3	(30.0)
Deworming										
No	406	(75.2)	85	(75.2)	92	(73.6)	28	(75.7)	6	(60.0)
Yes	134	(24.8)	28	(24.8)	33	(26.4)	9	(24.3)	4	(40.0)
Tetanus Diphtheria injection										
No	365	(67.6)	77	(68.1)	84	(67.2)	26	(70.3)	6	(60.0)
Yes	175	(32.4)	36	(31.9)	41	(32.8)	11	(29.7)	4	(40.0)
Sex of newborn										
Female	257	(47.6)	57	(50.4)	53	(42.4)	18	(48.7)	5	(50.0)
Male	283	(52.4)	56	(49.6)	72	(57.6)	19	(51.3)	5	(50.0)
Facility level										
Lower	294	(54.4)	48	(42.5)	56	(44.8)	17	(45.9)	5	(50.0)
Higher	246	(45.6)	65	(57.5)	69	(55.2)	20	(54.1)	5	(50.0)
Total	540	(65.5)	113	(13.7)	125	(15.2)	37	(4.5)	10	(1.2)

*BP* blood pressure, *HT* hypertension

### The association between redefined BP category and adverse perinatal outcome

**Fig. 2:**
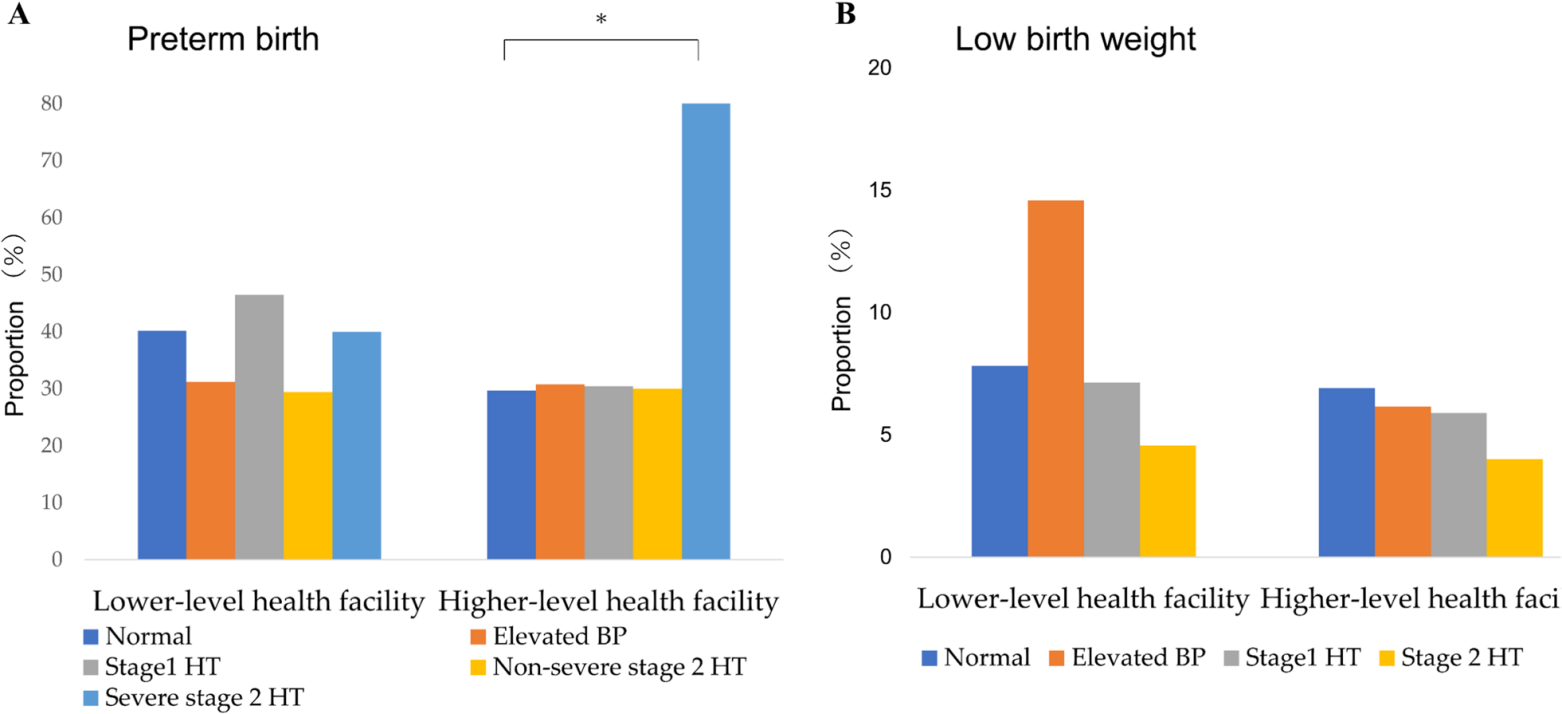
Incidence of adverse outcomes according to the redefined ACC/AHA blood pressure category. *BP* blood pressure, *HT* hypertension. *P < 0.05

The incidence of preterm birth and LBW was 166 (39.5%) and 35 (8.3%) at lower-level facilities and 124 women (30.6%) and 26 (6.4%) at higher-level facilities, respectively. The proportion of preterm births or LBW was not consistently higher in the group with abnormal BP than that in the normal group at lower-level facilities (Fig. 2). At the higher-level, women with severe stage 2 hypertension had remarkably more preterm birth, whereas the women with other abnormal BP categories showed similar proportions to normal women. For LBW at the higher-level facilities, dramatic differences by the BP category could not be found. At the lower-level facilities, both the proportion of preterm birth and LBW were equally low across all the BP categories. Tables 2 and 3 show the association between the redefined BP categories and preterm birth and LBW, respectively, at the facility level. For the lower facilities, the best GLM adjusted women’s anemia for preterm birth and deworming and the gestational week at delivery for LBW. No significant associations between BP categories and preterm birth were identified (elevated BP: adjusted odds ratio [AOR]:0.67; 95% confidence interval [CI]:0.35–1.30, stage1 hypertension: AOR:1.29; 95% CI 0.72–2.29, Non-severe stage 2 hypertension: AOR:0.62; 95% CI 0.21–1.82, Severe stage 2 hypertension: AOR:0.94; 95% CI 0.15–5.74). Similarly, there was no significant relationship between each BP category and LBW (elevated BP: AOR:2.12; 95% CI 0.84–5.32, stage1 hypertension: AOR:0.93; 95% CI 0.31–2.83, stage 2 hypertension: AOR:0.64; 95% CI 0.08–5.04). Regarding the higher facilities, ANC visit numbers for preterm birth and gestational weeks at delivery for LBW were included in the best GLM. The odds ratio of preterm birth was 10.94 times higher in the group of severe stage 2 hypertension than normal BP group, though no difference was found between the group of other BP categories (AOR:10.94; 95% CI 1.08–110.93). Regarding LBW, no significant difference was observed between redefined BP categories and normal BP (elevated BP: AOR:0.83; 95% CI 0.27–2.59, stage1 hypertension: AOR:0.81; 95% CI 0.26–2.52, severe stage 2 hypertension: AOR:0.50; 95% CI 0.06–3.98).

**Table 2:** Bivariate and multivariable logistic regression model of preterm birth (*N* = 825)

Variables	Lower-level facility (*n* = 420)				Higher-level facility (*n* = 405)			
	COR^a^	(95% CI)^b^	AOR^c^	(95% CI)	COR	(95% CI)	AOR	(95% CI)
Normal BP^d^	Ref		Ref		Ref			
Elevated BP	0.68	(0.35–1.30)	0.67	(0.35–1.30)	1.05	(0.58–1.91)	1.18	(0.63–2.18)
Stage1 HT^e^	1.29	(0.73–2.30)	1.29	(0.72–2.29)	1.04	(0.58–1.85)	1.10	(0.60–2.02)
Non-severe stage 2 HT	0.62	(0.21–1.81)	0.62	(0.21–1.82)	1.02	(0.38–2.75)	0.91	(0.33–2.50)
Severe stage 2 HT	0.99	(0.16–6.04)	0.94	(0.15–5.74)	9.48	(1.04–86.27)	10.94	(1.08–110.93)

The higher-level facility includes hospitals and the lower-level facility includes dispensaries and health centers. The best model for predicting preterm birth was selected by backward stepwise model selection using the lowest Akaike’s information criterion (AIC). The full model included variables of maternal blood pressure, anemia, obesity and age, preventive services and newborn sex. At the lower-level facility, the best model adjusted women’s anemia. At the higher-level facility, the best model adjusted ANC visit numbers

a: Crude odds ratio

b: 95% confidence interval

c: Adjusted odds ratio

d: Blood pressure

e: Hypertension

**Table 3:** Bivariate and multivariable logistic regression model of low birthweight (*N* = 825)

Variables	Lower-level facility (n = 420)				Higher-level facility (*n* = 405)			
	COR^a^	(95% CI)^b^	AOR^c^	(95% CI)	COR	(95% CI)	AOR	(95% CI)
Normal BP^d^	Ref		Ref		Ref			
Elevated BP	2.01	(0.81–4.99)	2.12	(0.84–5.32)	0.88	(0.29–2.72)	0.83	(0.27–2.59)
Stage1 HT^e^	0.91	(0.30–2.73)	0.93	(0.31–2.83)	0.83	(0.27–2.55)	0.81	(0.26–2.52)
Stage 2 HT	0.56	(0.07–4.36)	0.64	(0.08–5.04)	0.56	(0.07–4.40)	0.50	(0.06–3.98)

The higher-level facility includes hospitals and the lower-level facility includes dispensaries and health centers. The best model for predicting preterm birth was chosen using the lowest Akaike’s information criterion (AIC). The full model included variables of maternal blood pressure, anemia, obesity and age, preventive services, newborn sex and gestational week at delivery. At the lower-level facility, the best model adjusted deworming and the gestational week at delivery. At higher-level facilities, the best model adjusted gestational weeks at delivery

a: Crude odds ratio

b: 95% confidence interval

c: Adjusted odds ratio

d: Blood pressure

e: Hypertension

## Discussion

This study aimed to examine the association between revised ACC/AHA BP categories in pregnancy and preterm birth and LBW. The results demonstrated that only severe stage 2 hypertension, which was 160/110 mm Hg or higher, was significantly related to preterm birth compared to normal BP at the higher-level facility (Table 2). The incidence of LBW has presented no significant difference between every abnormal BP category and normotension in both the lower- and higher-level facilities in this study (Table 3).

Thus, this study revealed lower BP thresholds in pregnancy, such as elevated BP and stage 1 hypertension, were not associated with preterm birth and LBW. This finding was consistent with a systematic review of meta-analyses of 12 studies using BP of more than 20 gestational weeks [13]. They showed no relationship between elevated BP and stage 1 hypertension and preterm birth or small for gestational age. In contrast, significant associations were presented by other retrospective studies using the BP of before 20 weeks of gestation as the criteria for chronic hypertension [3, 9, 10, 14]. Our study did not consider chronic hypertension because of the purpose of evaluating the association between BP itself and adverse perinatal outcomes. BP measurement period and frequency varied by studies. Hence, further studies considering the characteristics of BP changes with gestational weeks are needed for more evidence.

In this study, the prevalence of elevated BP, stage 1 hypertension, non-severe stage 2 hypertension and severe stage 2 hypertension was 13.7%, 15.2%, 4.5%, and 1.2%, respectively (Table 1). This prevalence is similar to a study that was conducted in LMICs and classified women, as our study used maximum BP during pregnancy [33]. Thus, the use of elevated BP or stage 1 hypertension as a diagnostic cutoff makes healthcare providers provide additional antenatal reviews for 29% or 15% of women in addition to current women. This is undoubtedly a huge burden for healthcare facilities in low-resource settings because they have been suffering from a shortage of human resources for a long time [34]. Overall, our findings recommend retaining the current BP threshold to screen high-risk groups for preterm birth and low birthweight until more evidence is available.

This study demonstrated that even the current threshold (non-severe and severe stage 2 hypertension) had no relationship with adverse outcomes, except for preterm birth at higher-level health facilities. This can be explained by two reasons. The first reason is the small sample size. The majority of previous studies analyzed data from more than 10,000 women in high-income settings though our sample size was less than 1000 [3, 9–11, 14, 33]. Our study inclusion criteria, as at least one ANC and delivery at the WIRE facilities, made it difficult to investigate more women. In our research site, delivery at the parent’s home and moving outside of the WIRE coverage area often occurred. The second reason may be fewer baseline characteristics to adjust OR compared with previous studies. The causes of preterm birth and low birthweight are multifactorial [35, 36]. Other studies included socio-demographics, lifestyle and clinical characteristics for their analysis [9, 11, 14]. This study had only basic maternal data based on routine ANC and delivery records of the MCH book.

Most research on the association between ACC/AHA’s redefined classification of BP and perinatal adverse outcomes has been conducted in well-resourced settings and very little in LMICs [12, 13, 16, 37]. In low-resource settings, screening and prevention of adverse maternal and perinatal outcomes are essential because of the difficulty in treatment after it occurs. Our findings, therefore, help policymakers decide the evidence-based criteria and interventions for hypertension in pregnancy in low-resource settings. However, further studies are also needed in two aspects. First, this study examined the association with adverse outcomes without considering changes in BP during pregnancy as in most previous studies. In the study area, the delay of the first ANC visits is still a problem, although the Kenyan government recommends it before trimestral weeks of gestation [38]. More than 90% of women in this study had taken the first ANC after 20 weeks of gestation which is the deadline to detect chronic hypertension. Furthermore, more than 80% of women have received less than four ANC, although more than four times are recommended [29]. Most of them have had only one ANC visit in this study. Hence, prospective studies that regularly measure BP over time to examine BP characteristics by gestational week are needed for accurate evaluation. Secondly, our study focused on neonatal outcomes because they are critical for avoiding neonatal and infant death and health burdens in the future in LMICs. Many studies have demonstrated that women with elevated BP and stage 1 hypertension have increased risks for adverse maternal and perinatal outcomes; they included preeclampsia and perinatal morbidity and mortality, which were not evaluated in this study [9, 10, 16, 37]. Therefore, further studies are required to evaluate the association between new BP categories and various clinically critical outcomes for the final decision of criteria in pregnancy.

## Conclusion

The results revealed that the redefined lower threshold for abnormal BP in pregnancy had no association with preterm birth or low birthweight in low-resource settings. In contrast, stage 2 hypertension partly showed a relationship with adverse outcomes. Therefore, we recommend retaining the current criteria to identify high-risk pregnant women for preterm birth and low birth weight. However, numerous studies have presented that lower threshold such as elevated BP and stage 1 hypertension increase the risk of adverse maternal, perinatal, and neonatal outcomes. In addition, studies that consider the BP characteristics during pregnancy are insufficient. Therefore, further studies considering various adverse outcomes and changes in BP during pregnancy are needed to determine the hypertension criteria for pregnancy based on evidence.

## Data Availability

All the data analysed in this study are included in this article.

## Abbreviations

BP: Blood pressure
sBP: Systolic blood pressure
dBP: Diastolic blood pressure
ACC: American College of Cardiology
AHA: American Heart Association
LMICs: Low- and middle-income countries
LBW: Low birthweight
WIRE: Women and Infant Registration
ANC: Antenatal care
PNC: Postnatal care
MCH: Maternal and child health
LLITN: Long-lasting insecticide treated net
TD: Tetanus Diphtheria
GLM: Generalized linear model
OR: Odds ratio
AIC: Akaike’s information criterion
COR: Crude odds ratio
AOR: Adjusted odds ratio
CI: Confidence interval
KEMRI: Kenya Medical Research Institute
SERU: Scientific Ethical Review Unit
